# Antibacterial and Anti-Inflammatory Potential of Polyherbal Formulation Used in Chronic Wound Healing

**DOI:** 10.1155/2021/9991454

**Published:** 2021-07-06

**Authors:** Ilona Mandrika, Somit Kumar, Baiba Zandersone, Sujith Subash Eranezhath, Ramona Petrovska, Iveta Liduma, Arnolds Jezupovs, Valdis Pirags, Tatjana Tracevska

**Affiliations:** ^1^Faculty of Medicine, University of Latvia, Riga 1004, Latvia; ^2^Latvian Biomedical Research and Study Centre, Riga 1067, Latvia; ^3^AVP Research Foundation, Coimbatore 641045, India

## Abstract

**Objective:**

Polyherbal formulations *Jathyadi Thailam* and *Jatyadi Ghritam* (*JT*) are used in Indian traditional medicine for diabetic chronic wounds, fistula, fissure, eczema, and burn management. We aimed to investigate the antibacterial and anti-inflammatory properties of crude hexane and ethanol extracts of *JT* formulations.

**Methods:**

Antibacterial activity of *JT* extracts was tested to estimate minimum inhibitory concentrations (MICs) against nine reference bacterial strains, including one methicillin-resistant *Staphylococcus aureus* (MRSA) and multidrug-resistant (MDR) *Pseudomonas aeruginosa*, and clinical strains of methicillin-susceptible *S.aureus* (MSSA), all involved in diabetic foot infection. The anti-inflammatory activity of plant extracts was evaluated in LPS-treated macrophage cells by measuring the mRNA levels and secretion of inflammatory mediators.

**Results:**

The antibacterial activity of *JT* extracts was higher against Gram (+) bacteria, with the MICs varying from 1.95 to 62.5 mg/mL. Gram (−) bacteria were only susceptible to ethanol extracts of *JT*. Plant extracts were found to be the most active against the reference and clinical strains of MSSA, MRSA, and biofilm-forming *S. epidermidis*. *JT* extracts efficiently inhibited in a dose-dependent manner the mRNA expression and protein secretion of proinflammatory cytokines IL-6 and IL-1*β*, and chemokines MCP-1 and CXCL10 in LPS-challenged macrophages.

**Conclusion:**

In the present study, we have shown that extracts of *JT* formulations possess potent antibacterial and anti-inflammatory properties that could be involved in chronic wound healing activity and has the potential to be used as external add-on therapy in the management of multidrug-resistant bacterial infections at the wound.

## 1. Introduction

Diabetes is a chronic metabolic disease characterized by prolonged hyperglycemia, which can lead to eyes, kidneys, nerves, blood vessel damage, and nonhealing wounds [1, 2]. The normal wound healing process involves overlapping phases of hemostasis, inflammation, proliferation, and remodelling and requires a coordinated interplay between macrophages, fibroblasts, keratinocytes, epithelial cells, and signalling molecules. Macrophages play a critical role in the inflammatory phase of wound repair by killing pathogens, clearing damaged tissues, and producing growth factors that induce angiogenesis, collagen deposition, and wound closure [3]. During impaired healing of wounds associated with diabetes, it exhibits prolonged accumulation of M1-polarized macrophages associated with elevated levels of proinflammatory cytokines and reduced levels of various growth factors. It has been demonstrated that IL-1*β*, TNF-*α*, IL-6, and MCP-1 levels are sustained at high levels in chronic wounds of diabetic mice and humans [4].

Further, the diabetic wound environment with the resident cells and their secreted signalling molecules could be significantly influenced by the bacterial colonisation in the wound. It has been shown that diabetic mouse wounds inoculated with *Pseudomonas aeruginosa* biofilm displayed higher levels of IL-1*β* and IL-6 than control wounds even at 4 weeks postwounding [5]. Diabetic foot ulcer infections (DFI) are mostly polymicrobial, involving both aerobic and anaerobic bacteria that grow in synergy [6, 7]. Recent studies have revealed that Gram (+) cocci, mostly *Staphylococcus aureus*, are the main microorganisms responsible for DFI in occidental countries, but in Asia and Africa, Gram (-) bacilli are predominant. Among the *S. aureus* pathogen, methicillin-resistant *S. aureus* (MRSA) was found in 11% of the DFI isolates [8]. Multidrug-resistant bacterial infections are growing worldwide as a serious problem and this urges the immediate need for the search for alternatives.

The use of medicinal plants and herbs with oils or clarified butter as oleaginous products is a common practice in Indian traditional medicine. These practices have got enshrined in the traditional books as medicated oil (Thailam) and medicated clarified butter or ghee (Ghritam) [9, 10]. This study looks into the prospects of an Ayurveda-based polyherbal formulation, *Jatyadi* widely used in traditional medicine for chronic wounds (including diabetic wounds), ulcers, eczema, scalds, and burns healing as a topical application [11, 12]. The pharmacopoeia version of *Jatyadi (Jatyadi Ghritam)*, as in Ayurveda Formulary of India (AFI), consists of 11 herbs along with honey bee wax (*Sikta*) and copper sulfate (*Tuttha*) [10]. *Jatyadi Thailam (JT*_AFI_) is also prepared in a sesame oil base instead of clarified butter as mentioned in the pharmacopoeia version of *Jatyadi Ghritam,* without changing the herbal ingredients. *JT*_AFI_ has shown significant clinical efficacy in healing diabetic wounds after application for 10 days, with a follow-up of 45 days showing no recurrence [13]. Another clinical case of acute erythroderma showed complete remission in lesions after three months of external application of *JT*_AFI_ along with other parenteral drugs [14]. A very preliminary attempt has been made to understand the mechanism of action of *JT Thailam* through healing in excision wound model in rats [15] and *JT Ghritam* in diabetes-induced rats [16]. Interestingly, there is another traditional version of this formulation, namely, *Jatyadi Thailam (JT*_YG_) itself, which is used for similar indications in South India. The composition of this formulation (*JT*_YG_) is different from *JT*_AFI_ and is referenced in the Yogagrantha text [17]. *JT*_*YG*_ is composed of 13 herbs fortified in coconut oil. The *JT*_YG_ formulation has not been evaluated using contemporary methods, even though it is used in clinical practice for healing chronic wounds [18].

The aim of this study was to evaluate the biological activities of hexane (*JT*_AFI_-Hex, *JT*_YG_-Hex) and 90% ethanol (*JT*_AFI_-EE, *JT*_YG_-EE) extracts of herbal fractions of *JT*_AFI_ and *JT*_YG_ formulations *in vitro.* Antibacterial study on *JT* extracts was done through broth microdilution method to estimate its MICs against nine reference Gram (+) and Gram (−) bacterial strains and on clinical strains of MSSA, all involved in diabetic foot infection. The effects of plant extracts on proinflammatory cytokine and chemokine gene expression and protein secretion were examined using lipopolysaccharide- (LPS-) stimulated human THP-1 macrophage cells as a model to understand the network of reactions at the molecular plane during the inflammatory phase of diabetic wounds.

## 2. Materials and Methods

### 2.1. Collection and Authentication of Plant Material

Fresh plant materials were collected in and around Palghat District, Kerala, India, and the dried herbs were collected from respective herb dealers across India. The samples were then authenticated at Fischer Herbarium (FRC) at the Institute of Forest Genetics and Tree Breeding, Coimbatore, recognised by the Royal Botanical Gardens of UK and certified by Dr. Kunjikanan. The specimens were deposited in the FRC with voucher numbers for all individual herbs, except *Berberis aristata*, covered from 24222 to 24242. The details of the herbs and voucher numbers are summarized in Table 1 and Table 2. *Berberis aristata* root was identified based on the phytopharmacognostic method as stated in API [19].

### 2.2. Preparation of Plant Material and Extraction

All the plant materials were air-dried under shade and pulverized. 1000 g of both variants of *JT* herb mixtures was sequentially extracted in 4 L of nonpolar solvent hexane (Hex) and polar solvent 90% ethanol (EE) for 6 h at room temperature by hot percolation method using Soxhlet apparatus. Hexane and 90% ethanol extracts were concentrated up to 200 mL and 250 mL, respectively, and subjected to qualitative and quantitative analysis for the phytochemical profile. The concentrated extracts were further evaporated, and EE extract was lyophilized to remove the last traces of the water. The obtained yields were 2.14% (w/w) for *JT*_YG_-Hex, 3.58% (w/w) for *JT*_YG_-EE (semisolid), 0.72% (w/w) for *JT*_AFI_-Hex, and 3.33% (w/w) for *JT*_AFI_-EE (semisolid).

Stock solutions of 0.1 g/mL of extracts with 90% of final DMSO concentration have been made for cell culture analysis and stock solutions of 0.5 g/mL with 50% of final DMSO and 0.25% Tween 20 concentrations have been made for antibacterial studies.

### 2.3. Phytochemical Analysis of the *JT* Extracts

Extracts were tested initially for the presence of phytochemicals qualitatively according to standard methods such as precipitation or colouration tests as described by Evance [20] and further quantitative estimation was done only for the detected phytochemicals. Quantification of the total phenolic and tannin content of extracts was done by the Folin-Ciocalteu method according to [21, 22]. The concentrations of the phenols and tannins were estimated from gallic and tannic acids calibration curves, respectively. The aluminium chloride colorimetric method with a quercetin as a standard was used for the determination of the total flavonoid content of extracts as previously described [23]. The total anthocyanin content of extracts was determined by the pH differential method according to [24]. The Liebermann–Burchard test was used for determination of sterols, with cholesterol used as the standard as previously described [25]. The quantification of terpenoids, cardiac glycosides, and saponins was performed by gravimetric methods according to [26–28]. Quantitative determination of alkaloids and proteins was according to the methodology by Harborne [26] and Lowry [29].

### 2.4. Gas Chromatography-Mass Spectrometry (GC-MS) Analysis

Four millilitres of 0.5 N methanolic sodium hydroxide was added to the 150 mg of *JT*-Hex extracts and heated on a steam bath until the fat globules went into solution. After 5 min, 5 mL of BF3-methanol was added to the flask and the mixture was boiled for 2 min. A saturated sodium chloride solution was added to the flask to float the methyl esters. They were transferred with a syringe to a separatory funnel and 20 mL of petroleum ether (b.p. 30–60°C reagent grade redistilled) was added. The layers were separated by 1 min vigorous shaking. The petroleum ether layer was drained through filter paper and the solvent was then evaporated on a 60°C water bath. A 1 mL aliquot ester of the *JT*-Hex extracts was subjected to analysis by GC-MS.

GC-MS analysis was conducted on an Agilent 7890A GC coupled with a 5975 mass selective detector (Agilent Technologies, USA). Samples were separated on a DB-5MS capillary column (30 m length *x* 0.25 mm diameter *x* 0.25 *µ*m film thickness). The oven temperature was held at 40°C for 5 min and raised to 280°C at a rate 5°C/min. Pure helium was used as a carrier gas at a constant flow rate of 1 mL/min. Diluted samples in methanol of 3 *µ*L were injected in the split mode with a split ratio of 50 : 1. The chemical composition of the extracts was identified from the chromatograms and mass spectra using the database of NIST08 Library.

### 2.5. Bacterial Strains

The following reference and clinical bacterial strains were used in this study: *S. aureus* ATCC 25923, *S. aureus* (MRSA) ATCC 33592, *S. epidermidis* RP62 A, *Enterococcus faecalis* 29212, *Escherichia coli* ATCC 25922, *Pseudomonas aeruginosa* ATCC 27853, multidrug-resistant (MDR) *P. aeruginosa* ATCC BAA-2108, *Proteus mirabilis* ATCC 29245, and *Klebsiella pneumoniae* ATCC 33495. All the reference strains were obtained from the microbiology laboratory at Riga East University Hospital (REUH). The microorganisms were revived for bioassay by subculturing in fresh nutrition Trypticase soy broth for 24 h before use. The clinical material was collected by a sterile cotton swab from diabetic foot ulcers from DFI patients admitted to REUH. Collection of clinical samples from diabetic foot wounds for bacterial isolates was approved by the ethics committee at the Institute of Cardiovascular and Regenerative Medicine, University of Latvia. Four *S. aureus* cultures were isolated and identified microbiologically up to a species level at the REUH microbiology laboratory using the BBL Crystal system (Becton, Dickinson, UK). Susceptibility to a panel of antimicrobials was tested according to Clinical and Laboratory Standard Institute guidelines [30] by the agar disk diffusion test and the E-test using Mueller–Hinton (MH) agar (Becton Dickinson, UK) against the following panel of antibacterials: penicillin, gentamicin, cefazolin, erythromycin, clindamycin, vancomycin, ciprofloxacin, and trimethoprim-sulfamethoxazole. Methicillin resistance was tested by a cefoxitin disk on an agar screen plate. Plates inoculated with clinical *S. aureus* cultures were incubated for 20–24 h. The antibiogram of four *S. aureus* strains showed susceptibility to methicillin (cefoxitin).

### 2.6. Broth Microdilution Method

The antibacterial efficacy of the *JT* extracts was tested by determining the minimum inhibitory concentration **(**MIC) by broth microdilution method as described by Balouiri [31] with modifications. Stock solutions of gentamicin (0.4 mg/mL) and streptomycin (1 mg/mL) were used as MIC controls in each experiment according to CLSI standards CLSI-2017-M02-A12 [30]. Unsupplemented MH broths with and without the bacterial inoculate were used as positive and negative controls, respectively, in each experiment. DMSO was used as vehicle control for the inhibitory effect on all bacterial strains. The twofold dilution series of diluent DMSO were made in each well (starting from 12.5% in the first well, 6.25% in the second well, and so forth). The concentration of DMSO at 6.25% in the second well was below the toxic level as tested separately and did not inhibit the growth of bacteria.

Twofold dilution series of each extract were prepared in MH broth (starting from a concentration of 250 mg/mL) and added to 96-well microtiter plate (100 *µ*L/well). Overnight grown fresh bacterial cells were suspended in sterile saline and adjusted to a turbidity of McFarland standard No 0.5, bacterial cell suspension containing 1.5 × 10^8^ colony-forming units (CFU/mL). The bacterial cell suspension was further diluted 1 : 100 in distilled water and aliquoted into a microplate 100 *µ*L per well. To calculate MIC, after 20 h of incubation at 35°C, 10 *µ*L aliquots of the appropriate dilution were withdrawn from the microtiter plate and inoculated on MH agar in a Petri dish and incubated for 24 h. Colony-forming units of each dilution were counted and compared to the control Petri dish inoculated 24 h earlier with 10 *µ*L of the same strain suspension for MIC determination. The MIC value was calculated manually and assumed the same as minimum bactericidal concentration when 99.9% inhibition of bacterial growth after inoculation in a Petri dish was observed [31]. Each extract was tested in triplicate for statistical reliability.

### 2.7. Cell Culture and Differentiation Induction

The human THP-1 monocyte cell line was obtained from ATCC (American Type Culture Collection). THP-1 cells were maintained in RPMI-1640 medium containing 10% fetal bovine serum and antibiotics and incubated in 5% CO_2_ at 37°C. THP-1 monocytes were stimulated to differentiate into macrophages with phorbol 12-myristate 13-acetate (PMA) (20 ng/mL) for 24 h. Medium was exchanged for PMA-free medium for 48 h before cells were used for experiments.

### 2.8. Cell Viability Testing

Cell viability was tested by Cell Counting Kit-8 (CCK-8) (Sigma-Aldrich, USA), according to the manufacturer's instructions. THP-1 cells (100 *µ*L) were seeded into a 96-well tissue culture plate at the density of 10^4^ cells per well and grown in the presence or absence of various concentrations (3–200 *µ*g/mL) of *JT* plant extracts. Wells with dead THP-1 cells were used as the negative control wells. CCK-8 solution (10 *µ*L) was added to the cells at 24 and 48 h and plates were incubated for 1 h at 37°C. The absorbance of each well was measured at 450 nm using a microplate reader Victor3^TM^ (Perkin Elmer, USA). Wells without cells but containing medium were used as a blank value that was subtracted from all values. Each assay was performed in triplicate.

### 2.9. RNA Extraction and Quantitative Real-Time Polymerase Chain Reaction (qRT-PCR)

Total RNA was isolated from cultured cells using TRIzol reagent (Sigma, USA), according to the manufacturer's protocol. First-strand cDNA was synthesized from 2 *µ*g RNA using oligo (dT) primers with Revert Aid H Minus kit (Thermo Fisher Scientific, USA). qRT-PCR was performed using Absolute Blue SYBR green Master Mix reagent (Thermo Fisher Scientific, USA) with a ViiA7 real-time PCR detection system (Applied Biosystems, USA). The primer pairs used for the gene amplifications are listed in Table 3 mRNA levels were quantified and normalized to levels of reference gene *RPS29* using the 2^−ΔΔCt^ method and presented as relative expression compared with values of untreated cells.

### 2.10. Quantification of Cytokines and Chemokines

Conditioned media were obtained from untreated and *JT* extract-treated THP-1 macrophages. The concentrations of the IL-6, IL-1*β*, MCP-1, and CXCL10 biomolecules were measured by Luminex Multiplex immunoassay (R&D Systems, USA) according to the manufacturer's instructions and analyzed on a Luminex 200 (Luminex Corporation, USA). At least three independent repetitions in duplicate were made per sample. Concentrations of the analytes were quantified with five parameters logistic (5-PL) curve fit and expressed in pg/mL.

### 2.11. Statistical Analysis

Statistical analysis of MIC data was done using chi-square test. The median value for each MIC was calculated. Gene expression results are expressed as mean ± standard deviation (SD) of the indicated number of experiments. Student's *t*-test was used to assess the statistical significance of differences. Significant differences were assumed for *p* < 0.05. The statistical analysis was performed using GraphPad Prism 5.0 software (GraphPad Software Inc, USA).

## 3. Results

### 3.1. Phytochemical Evaluation of *JT* Extracts

The quantitative phytochemical screening of obtained *JT* plant extracts revealed the presence of phenols, proteins, tannins, sterols,and terpenoids in *JT*_YG_-EE, *JT*_AFI_-EE, and *JT*_AFI_-Hex extracts, as shown in Table 4. All *JT* extracts had flavonoids with the highest content in *JT*_YG_-EE extract. Both *JT*_YG_-EE and *JT*_AFI_-EE were rich in proteins. Alkaloids were detected only in *JT*_AFI_-EE and saponins only in *JT*_YG_-EE extracts. Glycosides were found to be present only in hexane extracts of both *JT* formulations with the higher concentration in *JT*_YG_*-*Hex. Anthocyanins were present only in *JT*_YG_ extracts. Overall, *JT*-EE extracts had a higher content in phenols, proteins, tannins, sterols, and flavonoids.

### 3.2. Chemical Composition of *JT* Extracts

The presence of chemical components in the *JT* hexane extracts was analyzed by GC-MS. The analysis of *JT*_YG_-Hex extract has shown the presence of 36 peaks in the gas chromatogram and 21 components corresponding to the major peaks have been determined and are listed in Table 5. Major chemical compounds were 9,12-octadecadienoic acid or linoleic acid (24.86%), n-hexadecanoic acid or palmitic acid (17.17%), cis 13- octadecenoic acid (15.19%), cis-13,16-docosadienoic acid (5.29%), ar-turmerone (2.49%), alpha-turmerone (2.03%), beta-turmerone or curlone (1.84%), 9-octadecenoic acid (Z) methyl ester or oleic acid (1.46%), phytol (1.61%), and squalene (1.84%), while other compounds were present in minor quantities with peak areas ranging from 0.09 to 1.61%.

Gas chromatogram analysis of *JT*_AFI_-Hex extract revealed 33 distinct peaks and the 25 components corresponding to the major peaks have been determined and listed in Table 6. Major chemical compounds were 6-octadecenoic acid (27.36%), n-hexadecanoic acid (10.17%), 3-dibenzofuranamine (13.24%), octadecane (5.67%), fumaric acid, 2, 4-dimethyl pent-3-yl isohexyl ester (5.36%), fumaric,3,5-dichlorophenyl isohexyl ester (4.99%), 9,12-octadecadienoic acid (Z, Z) (3.12%), and phytol (2.23%), while other compounds were present in minor quantities with peak areas ranging from 0.321 to .97%.

### 3.3. Antibacterial Activity of *JT* Extracts on Reference Bacterial Strains

The antibacterial properties of *JT* extracts were elucidated on four Gram (+) (*S. aureus* ATCC 25923, MRSA ATCC 33592, *S. epidermidis* RP62 A, and *E. faecalis* 29212) and five Gram (−) (*E. coli* ATCC 25922, *P. aeruginosa* ATCC 27853, MDR *P. aeruginosa* ATCC BAA-2108, *P. mirabilis* ATCC 29245, and *K. pneumoniae* ATCC 33495) reference bacterial strains. The results of MIC as determined by broth microdilution method are shown in Table 7. In general, MIC values ranged from 1.95 to 62.5 mg/mL. The extracts at bactericidal concentrations higher than 125 mg/mL were considered as having no inhibition since the bacterial growth was influenced by the diluent 12.5% DMSO.

All *JT* extracts showed better inhibitory activity for Gram (+) bacteria compared to Gram (−) (Table 7). When the cases of inhibition (MIC determined) were compared to the cases without inhibition, 100% of Gram (+) and only 40% of Gram (−) bacterial strains had cases of inhibition by extracts studied. Both *JT*_YG_-Hex and *JT*_YG_-EE extracts showed lower MIC for Gram (+) bacterial reference strains (median 11.7) than MIC for Gram (−) strains (median ≥125.0). Similarly, both *JT*_AFI_-EE and *JT*_AFI_-Hex extracts were also more active against Gram (+) (median 31.25) compared to MIC for Gram (−) (median 93.75) reference strains. The overall efficacy of *JT*_YG_ formulation extracts (median 11.7) was higher than that of *JT*_AFI_ formulation extracts (median 31.25).

In order to investigate the best efficacy for Gram (+) bacteria, the MICs were further compared between the extracts. The lowest MIC was detected for biofilm-forming *S. epidermidis* RP62 A strain by *JT*_YG_-EE and *JT*_YG_-Hex extracts (1.95 mg/mL) followed by *JT*_AFI_-EE extract (15.6 mg/mL) and by *JT*_AFI_-Hex (31.25 mg/mL), as shown in Table 7. Notably, the results indicated that all four extracts were active against MRSA ATCC 33592 strain, with the highest inhibitory activity shown by *JT*_YG_-EE and *JT*_AFI_-EE extracts (15.6 mg/mL) (Table 7).

Further, the overall inhibitory efficacy for Gram (+) and Gram (−) bacteria was compared between the extracts. Gram (-) bacteria were susceptible only to EE extracts, except for *P. aeruginosa* MDR ATCC BAA-2108. There was no significantly important difference (*p* = 0.527) in the frequency of inhibition between *JT*_YG_-EE (median 31.25) and *JT*_YG_-Hex (median 62.5). There was also no significantly important difference (*p* = 0.836) in the frequency of inhibition between *JT*_AFI_-EE (median 62.52) and *JT*_AFI_-Hex (median ≥125), between *JT*_YG_-EE (median 31.25) and *JT*_AFI_-EE (median 31.25), and between *JT*_YG_-Hex (median 62.5) and *JT*_AFI_-Hex extract (median 62.5) (Table 7).

### 3.4. Antibacterial Activity of *JT* Extracts on Clinical *S. aureus* Strains

Additionally, four *S. aureus* clinical strains, namely, 704, 526, 250, and 588, isolated from DFIs, were examined for antibacterial activity by broth microdilution method. The results of MICs are shown in Table 8. The values of MIC varied from 1.95 to 62.5 mg/mL among clinical strains and among extracts, except for *JT*_YG_-Hex extract (7.81 mg/mL). There was no difference in MIC between *JT*_YG_-EE (median 15.6) and *JT*_AFI_-EE (median 15.6) extracts and, similarly, between *JT*_YG_-Hex (median 62.5) and *JT*_AFI_-Hex extracts (median 62.5), with the EE extracts showing higher activity compared to that of Hex extracts. Our data showed that the frequency of inhibition was not significantly different between *S. aureus* clinical strains and the reference group (*p* = 0.590).

As a component of the original *JT*_AFI_ formulation, possessing antibacterial potential on the skin, the inhibitory effect of copper sulfate was additionally tested on *S. aureus* clinical strains by broth microdilution method. Copper sulfate stock solution was prepared at a concentration of 3.53 mg/mL, which is equal to that used in the original *JT*_AFI_ product (10.6 g/3 L of water). Our data indicate that MIC was achieved by copper sulfate already at concentration 0.88 *µ*g/mL, which is much lower than the concentration in the original *JT*_AFI_ product (Table 8).

### 3.5. Effects of JT Plant Extracts on Cell Viability

**Figure 1:**
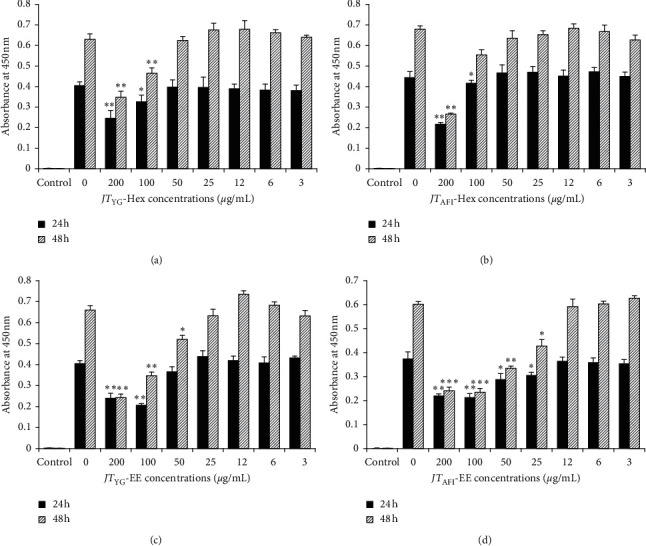
Effects of *JT*_YG_ and *JT*_AFI_ plant extracts on THP-1 cells viability. THP-1 macrophages were grown in the presence of indicated concentrations of *JT*_YG_-Hex (a), *JT*_AFI_-Hex (b), *JT*_YG_-EE (c), and *JT*_AFI_-EE (d) extracts for 24 and 48 h. Cell viability was measured using the CCK-8 solution. Data are a summary of three independent experiments performed in duplicate and expressed as mean ± SD. ^*∗*^*p* < 0.05, ^*∗*^^*∗*^*p* < 0.01, ^*∗*^^*∗*^^*∗*^*p* < 0.0001 are significantly different from untreated cells.

The effects of *JT* extracts on the viability of THP-1 macrophages were examined to determine the concentrations of the extracts that are nontoxic for the cells. As can be seen from Figure 1, no cytotoxic effect was observed following a 24 and 48 h treatment of the cells with both the *JT*_YG_-Hex and *JT*_AFI_-Hex at concentrations less than 50 *µ*g/mL and the *JT*_YG_-EE and *JT*_AFI_-EE at concentrations less than 12 *µ*g/mL. Therefore, for subsequent experiments, concentrations of 50 *µ*g/mL for *JT*_YG_-Hex and *JT*_AFI_-Hex and 10 *µ*g/mL for *JT*_YG_-EE and *JT*_AFI_-EE were used to induce a maximal response without causing cell toxicity. Exposure to vehicle control DMSO ranging from 0.2 to 0.003% had no effect on cell viability (data not shown).

### 3.6. JT Extracts Suppresses Gene Expression of Inflammatory Cytokines and Chemokines in THP-1 Macrophages

**Figure 2:**
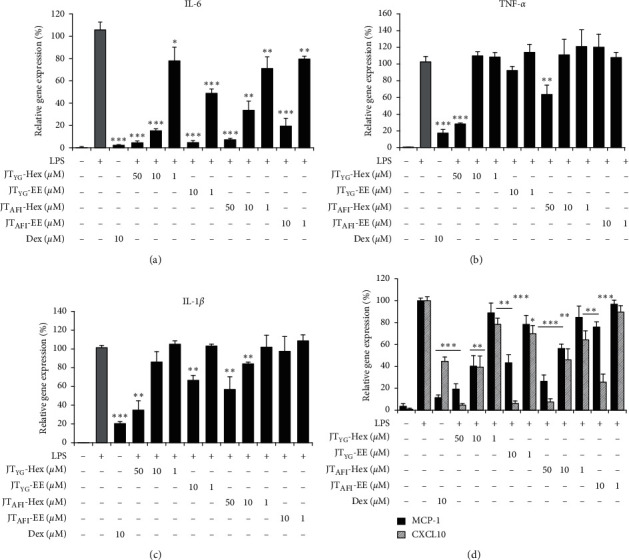
Suppression of proinflammatory cytokine and chemokine gene expression by *JT* extracts in THP-1 macrophages. Cells were treated 4 h with LPS (1 *µ*g/mL) in the presence or absence of various concentrations of *JT-*EE and *JT-*Hex extracts. The levels of cytokine IL-6 (a), TNF-*α* (b), IL-1*β* (c), and chemokine MCP-1 and CXCL10 (d) mRNA were determined by real-time PCR. Data were normalized to *RpS29* and values were calculated using the comparative (2^−ΔCt^) method. Results are mean ± SD from at least three independent experiments. ^*∗*^*p* < 0.05, ^*∗*^^*∗*^*p* < 0.01, ^*∗*^^*∗*^^*∗*^*p* < 0.0001 are significantly different from LPS-stimulated cells.

We quantified the expression levels of the proinflammatory cytokines IL-6, IL-1*β*, TNF-*α*, and chemokines-MCP-1 and CXCL10, which are chemoattractant factors involved in monocyte/macrophage and T-cell recruitment. THP-1 macrophages were stimulated with LPS (1 *µ*g/mL) with or without the addition of various concentrations of *JT*-Hex (50, 10, and 1 *µ*g/mL) and *JT*-EE (10 and 1 *µ*g/mL) plant extracts. Expression of each molecule at mRNA level was upregulated after 4 h stimulation with LPS in THP-1 macrophages (Figure 2). All extracts in a dose-dependent manner showed the most potent inhibition of IL-6, CXCL10 and MCP-1 gene expression. The addition of *JT*_YG_-Hex at 50, 10, and 1 *µ*g/ml resulted in significant reduction of LPS- stimulated IL-6 mRNA levels by 95%, 84% (*p* < 0.0001), 22% (*p* = 0.0019), CXCL10 mRNA levels by 95% (*p* < 0.0001), 61% (*p* = 0.0006), and 21% (*p* = 0.005) and MCP-1 levels by 81% (*p* < 0.0001), 60% (*p* = 0.005), and 11% (*p* = 0.096), respectively (Figures 2(a) and 2(d)). The addition of *JT*_AFI_-Hex at 50, 10 and 1 *µ*g/mL resulted in significant reduction of LPS-stimulated IL-6 mRNA levels by 92% (*p* < 0.0001), 66% (*p* = 0.0003), and 28% (*p* = 0.009), CXCL10 mRNA levels by 92% (*p* < 0.0001), 54% (*p* = 0.001), and 34% (*p* = 0.002) and MCP-1 levels by 74%, 44% (*p* < 0.0001), and 15% (*p* = 0.062), respectively (Figures 2(a) and 2(d)). The addition of *JT*_YG_-EE at 10 and 1 *µ*g/mL resulted in significant reduction of LPS-stimulated IL-6 mRNA levels by 95% and 51% (*p* < 0.0001), CXCL10 mRNA levels by 94 (*p* < 0.0001) and 30% (*p* = 0.003) and MCP-1 levels by 56% (*p* = 0.0002) and 22% (*p* = 0.011), respectively (Figures 2(a) and 2(d)). The addition of *JT*_AFI_-EE at 10 and 1 *µ*g/mL resulted in significant reduction of LPS-stimulated IL-6 mRNA levels by 80% (*p* < 0.0001) and 20% (*p* = 0.004), CXCL10 mRNA levels by 74% (*p* < 0.0001) and 10% (*p* = 0.058) and MCP-1 levels by 24% (*p* = 0.001) and 3% (*p* = 0.069), respectively (Figures 2(a) and 2(d)). A significant 65% (*p* = 0.0004) and 43% (*p* = 0.005) decrease in IL-1*β* mRNA and 72% (*p* < 0.0001) and 36% (*p* = 0.008) decrease in TNF-*α* mRNA was detectable at 50 *µ*g/mL for *JT*_YG_-Hex and *JT*_AFI_-Hex extracts, respectively (Figures 2(b) and 2(c)). However, only *JT*_YG_-EE at 10 *µ*g/mL was able to significantly (*p* = 0.001) inhibit IL-1*β* gene expression (Figure 2(c)). Exposure to 10 *µ*M dexamethasone used as positive control significantly decreased LPS-stimulated mRNA levels of all tested molecules (Figure 2). Exposure to vehicle control DMSO had no effect on cytokine expression in LPS- stimulated cells (data not shown).

### 3.7. Plant Extracts Suppresses Secretion of Inflammatory Cytokines and Chemokines in THP-1 Macrophages

**Figure 3:**
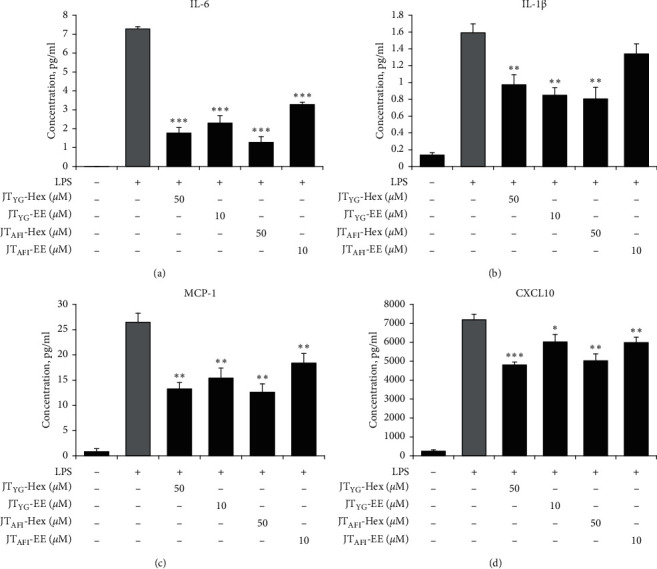
Suppression of proinflammatory cytokine and chemokine secretion by *JT* extracts in THP-1 macrophages. Supernatants from 4 h LPS and/or extracts-treated THP-1 macrophages were subjected to Luminex assay to detect proinflammatory cytokine IL-6 (a) and IL-1*β* (b) and chemokine MCP-1 (c) and CXCL10 (d) levels. Results are mean ± SD from three independent experiments. ^*∗*^*p* < 0.05, ^*∗*^^*∗*^*p* < 0.01, ^*∗*^*p* < 0.0001 are significantly different from LPS-stimulated cells.

As the next step, we measured cytokine levels secreted into the medium when THP-1 macrophages were exposed to LPS (1 *µ*g/mL) in the presence or absence of *JT*- Hex (50 *µ*g/mL) and *JT*- EE (10 *µ*g/mL) extracts. After 24 h, the culture medium was collected, and cytokine concentrations were measured with Luminex kit. All cytokine secretion significantly increased following macrophage treatment with LPS (Figure 3). *JT*_AFI_-Hex and *JT*_YG_-Hex extracts were able to significantly reduce the secretion of IL-6, IL-1*β*, MCP-1, and CXCL10 from macrophages (Figure 3). IL-6, MCP-1, and CXCL10 secretion was also significantly decreased by both *JT*-EE extracts (Figures 3(a), 3(c), and 3(d)). However, only *JT*_YG_-EE extract at 10 *µ*g/mL significantly decreased LPS-stimulated secretion of IL-1*β* (*p* = 0.005, Figure 3(b)).

## 4. Discussion

The reverse pharmacology approach can be applied to study formulations used in Ayurveda to understand their mode of actions. Few reports have offered clear underlying mechanisms of synergistic therapeutic actions of herbal ingredients [32]. This study attempts to understand the potentials of both traditional *JT* versions and the biological activities of hexane and ethanol extracts of herbal fractions of *JT*_AFI_ and *JT*_YG_ formulations *in vitro.*

So far, the wound healing mechanisms and efficacy of the *JT* formulation have been investigated using *in vivo* excision wound model and burn injury in rats [15, 33]. The authors reported faster wound healing process in rats treated with *JT* due to increased proliferation of fibroblasts, protein synthesis, enhanced collagen formation, reepithelization, and wound contracting ability. However, to the best of our knowledge, there are no studies on antimicrobial and anti-inflammatory potential of *JT*. Therefore, this study evaluated the antimicrobial and anti-inflammatory activities of ethanol and hexane extracts obtained from both *JT*_YG_ and *JT*_AFI_ formulations. *JT*_AFI_ formulation is composed of thirteen herbal ingredients along with copper sulfate fortified in oil. This study focuses only on the herbal fractions of both *JT* formulations. *JT*_AFI_ was evaluated without copper sulfate to understand the role of herbal combinatorics, as the efficacy of copper sulfate for its antimicrobial [34] and wound healing activity is well established [35].

Recent studies using molecular methods such as 16S rRNA sequencing on diabetic ulcers revealed much higher levels of microbial diversity, especially more anaerobes and Gram (−) species than those previously known through culture-based methods [36]. Other bacteria involved in DFI in Europe, as revealed by conventional culturing, include *P. mirabilis, P. aeruginosa, Klebsiella* spp.*, E. coli*, and anaerobes [37]. Contrary to this, in warmer countries (particularly in Asia and Africa), aerobic Gram (−) bacilli, especially *P. aeruginosa,* are more often the cause of DFIs. The antibacterial activity of *JT* extracts was evaluated against both Gram (+) and Gram (−) reference bacterial strains and against four *S. aureus* strains isolated from clinically infected diabetic foot ulcers. We observed that all *JT* extracts showed better inhibitory activity for Gram (+) bacteria than Gram (−) strains. Notably, Gram (-) bacteria were susceptible only to EE extracts, except in the case of bacterial strain *P. aeruginosa* MDR ATCC BAA-2108. This can be explained by the higher presence of phenols, tannins, sterols, and flavonoids in EE extracts. A direct correlation between the summary content of phenols and flavonoids and antibacterial activity of different plant extracts has been reported [38].

Interestingly, the most potent antibacterial activity of *JT* extracts was observed against the reference bacterial strains, namely, *S. aureus* (MRSA, MSSA) and *S. epidermidis*. Moreover, the extracts were effective against all four clinical strains of *S. aureus*, with the highest MIC value of 7.81 mg/mL for *JT*_YG_-Hex extract and varying in MICs from 1.95 to 62.5 mg/mL among clinical strains and *JT*_YG_-EE, *JT*_AFI_-EE, and *JT*_AFI_-Hex extracts. It has been shown that *S. aureus* is the most common pathogen among the Gram (+) bacteria isolated from infected diabetic foot wounds [39, 40]. Moreover, both *S. aureus* and *S. epidermidis* strains are able to form biofilms in a number of chronic wound states, including DFIs, which appear to impair wound healing by delaying wound reepithelization, reducing the effects of antimicrobials and immune response, and further increasing the infection of the wound [41].

In the recent past, treating DFIs has become more challenging due to the increased prevalence of multidrug-resistant pathogens, particularly MRSA. Interestingly, the prevalence of MRSA in DFI varies among countries with an increase in less developed countries [8]. Therefore, we have elucidated the antibacterial activity of *JT*-EE and *JT*-Hex extracts against two predominant multiresistant bacteria found in diabetic wounds-MRSA and MDR *P. aeruginosa*. All four extracts had the greatest potential value against MRSA, with the highest inhibitory activity by ethanol extracts of both *JT* formulations with MIC 15.60 mg/mL and following hexane fractions with MIC 31.25 mg/mL. However, only *JT*_YG_-Hex and *JT*_AFI_-EE extracts were able to inhibit the growth of the Gram (-) MDR *P. aeruginosa,* albeit with a higher MIC value of 62.5 mg/mL. The antibacterial effect of *JT* extracts found in this study could be one of the mechanisms, which accelerates wound healing activity and thus supports its traditional use.

Such microbial infections and higher levels of biofilm in chronic wounds could influence the activity of the cells involved in wound healing and the composition of secreted cytokines and growth factors. Proinflammatory cytokines in the wound are required for initiating normal wound repair, but their increased expression levels are required only transiently. Moreover, both bacteria and their produced endotoxins can lead to the prolonged elevation of proinflammatory cytokines such as IL-1*β*, IL-6, and TNF-*α* and downregulation of the anti-inflammatory cytokines TGF-*β* and IL-10 in diabetic wounds, resulting in prolonged inflammation stage of wound healing [5]. The present study explored whether *JT* extracts were able to modulate inflammatory activity in LPS-challenged THP-1 macrophage cells. Our results showed that all *JT* plant extracts dose-dependently decreased IL-6, MCP-1 and CXCL10 gene expression and protein secretion in THP-1 macrophages in response to LPS; however, only *JT*_YG_-EE was able to significantly reduce the protein secretion and gene expression of IL-1*β* cytokine. Hexane extracts of both *JT* formulations also showed the potency to modulate TNF-*α* gene expression. The *JT* extracts could be able to normalize overexpressed cytokines in chronic wounds and, therefore, could shorten the prolonged inflammation.

In the present study, the phytochemical analysis and GC-MS showed the presence of a considerable number of bioactive compounds and phytochemicals like flavonoids, terpenoids, sterols, glycosides, and fatty acids in the *JT* plant extracts, which can contribute to their antibacterial and anti-inflammatory activities. It has been shown that saponins, flavonoids, and phenols possess potent anti-inflammatory and antifungal activity. Terpenoids exhibit antibacterial activity and play an active role in wound healing, increase the concentration of antioxidants in wounds and restore inflamed tissues [42]. Furthermore, the *JT*- Hex extracts were rich in ar-turmerone, *a*-turmerone, and *ß*-turmerone, which are the major compounds in the essential oils of turmeric from *Curcuma longa*. Several studies have demonstrated that turmeric essential oils possessed potent antibacterial activity against a wide range of bacteria such as *S.aureus, E.coli*, *P.aeruginosa*, and *B.subtilis* [43–45]. Other phytoconstituents in *JT* extracts, such as squalene from *Cynodon dactylon,* phytol from *Erythrina variegate* and *Azadirachta indica*, and stigmasterol derived from *Pongamia pinata* may also contribute to the biological effects of the *JT* extracts. Studies have demonstrated that squalene promotes remodelling and tissue repair response signals by inhibiting the nuclear factor-kappa B, inducible nitric oxide synthase, the production of proinflammatory cytokines, such as IL-1*β* and TNF-*α*, and stimulation of anti-inflammatory cytokines IL-10 and IL-4 [46, 47]. The biological activities of phytol and phytosterols can be attributed to anti-inflammatory activities through reduction of leukocyte and neutrophil migration, IL-1*β*, IL-6, and TNF-*α* levels and oxidative stress, as well as antibacterial effects [48–50].

*JT* extracts showed a considerable number of free fatty acids such as palmitic, oleic, linoleic, stearic acids, and their esters. Free fatty acids have shown a vital effect in the prevention of antibacterial growth by targeting the structure and function of bacterial cell walls and membranes and inflammatory process via modulation of nitric oxide and cytokine production at the wound site [51, 52].

## 5. Conclusions

This study provides evidence that the ethanol and hexane extracts of both *JT* formulations possess potent antibacterial activity against Gram (+) and, to a lesser extent, Gram (−) bacteria and play an essential role in the anti-inflammatory response to LPS in macrophages. So, the study provides a rationale for the synergistic contribution of various herbs in the use of *JT* in the management of nonhealing wounds.

## Figures and Tables

**Table 1:** Herbal ingredients used for *JT*_YG_ preparation.

Specimens No.	Botanical name	Family	Vernacular name	Specimen Acc No.	Part used	Amount used of herbs
1	*Aerva lanata* (L.) Juss.	Amaranthaceae	Cherula	24229	Whole plant	1 part
2	*Calycopteris floribunda* (Roxb.) Lam. ex. Poir.	Combretaceae	Pullani	24227	leaf	1 part
3	*Curcuma longa* L.	Zingiberaceae	Manjal	24235	Rhizome	1 part
4	*Cynodon dactylon* (L.) Pers.	Poaceae (Graminae)	Arugampul	24222	Whole plant	1 part
5	*Erythrina variegate* L. (= *Erythrina indica* Lam.)	Fabaceae	Muruku	24224	Leaf	1 part
6	*Glycyrrhiza glabra* L.	Fabaceae	Erati mathuram	24236	Root	1 part
7	Jasminum flexile Vahl	Oleaceae	Jasmine	24241	Leaf	1 part
8	*Murraya koenigii* (L.) Spreng.	Rutaceae	Karuvepu	24231	Leaf	1 part
9	*Nigella sativa* L.	Ranunculaceae	Karujeeragam	24242	Seed	1 part
10	*Oldenlandia corymbosa* L.	Rubiaceae	Parpadagam	24230	Whole plant	1 part
11	*Physalis minima* L.	Solanaceae	Njotta	24237	Whole plant	1 part
12	*Pupalia atropurpurea*	Amaranthaceae	Cherukadaladi	24238	Whole plant	1 part
13	*Vitex negundo* L.	Lamiaceae	Karunochi	24228	Leaf	1 part

**Table 2:** Herbal ingredients used for *JT*_AFI_ preparation.

Specimens No.	Botanical name	Family	Vernacular name	Specimen Acc No.	Part used	Amount used of herbs
1	*Azadirachta indica* A. Juss.	Meliaceae	Nimba	24223	Leaf	1 part
2	*Chrysopogon zizanioides* (L.) Roberty (*Vetiveria zizanioides* (L.) Nash)	Poaceae (Graminae)	Usira	24233	Root	1 part
3	*Berberis aristata*	Menispermaceae	Darvi	^*∗*^	Stem	1 part
4	*Curcuma longa* L.	Zingiberaceae	Nisa	24235	Rhizome	1 part
5	*Glycyrrhiza glabra* L.	Fabaceae	Yastimadhu	24236	Root	1 part
6	*Hemidesmus indicus* (L.) R. Br. ex. Schult	Apocynaceae	Sariva (black)	24234	Root	1 part
7	*Jasminum officinale* Linn.	Oleaceae	Jati	24241	Leaf	1 part
8	*Picrorhiza kurroa* Royle ex. Benth	Plantaginaceae	Katuka	24240	Root/rhizome	1 part
9	*Pongamia pinnata* (L.) Pierre	Fabaceae	Karanja	24232	Seed	1 part
10	*Rubia cordifolia* L.	Rubiaceae	Manjistha	24225	Root	1 part
11	*Trichosanthes dioica* Roxb.	Cucurbitaceae	Patola	24419	Leaf	1 part

^*∗*^Identified as per standards mentioned in API [19].

**Table 3:** Sequences for qRT-PCR primers used in this study.

Primer name	Sequence (5′- 3′)	Product length, bp
IL-6 Fw	AGACAGCCACTCACCTCTTCAG	132
IL-6 Rv	TTCTGCCAGTGCCTCTTTGCTG	

IL-1*β* Fw	CCACAGACCTTCCAGGAGAATG	131
IL-1*β* Rv	GTGCAGTTCAGTGATCGTACAGG	

TNF-*α* Fw	CCCAGGGACCTCTCTCTAATCA	116
TNF-*α* Rv	AGCTGCCCCTCAGCTTGAG	

MCP-1 Fw	AGAATCACCAGCAGCAAGTGTCC	98
MCP-1 Rv	TCCTGAACCCACTTCTGCTTGG	

CXCL10 Fw	GGTGAGAAGAGATGTCTGAATCC	134
CXCL10 Rv	GTCCATCCTTGGAAGCACTGCA	

RPS29 Fw	CAAGATGGGTCACCAGCAG	106
RPS29 Rv	ATATTTCCGGATCAGACCGT	

**Table 4:** Quantitative phytochemical evaluation of the *JT* extracts.

Phytochemical (%)	Extracts			
	*JT*_YG_-Hex	*JT*_YG_-EE	*JT*_AFI_-Hex	*JT*_AFI_-EE
Alkaloids	—^*a*^	—	—	6.17
Phenols	—	6.20	4.60	8.28
Proteins	1.40	11.56	4.32	12.96
Tannins	—	9.80	5.69	14.32
Sterols	—	11.30	9.80	4.02
Glycosides	16.70	—	2.82	—
Saponins	—	11.04	—	—
Terpenoids	—	17.16	18.51	9.93
Flavonoids	3.06	9.02	2.70	5.26
Anthocyanins	0.19	0.50	—	—

^*a*^Quantitative evaluation of phytochemicals was not done as preliminary qualitative tests showing absence of these phytochemicals.

**Table 5:** Biologically active chemical compounds of *JT*_YG_-Hex extract.

S. No.	Retention time, min	Active components	Peak area, %
1	42.529	Ar-Turmerone	2.49
2	42.767	Turmerone	2.03
3	44.349	Curlone (beta-turmerone)	1.84
4	45.518	Bicyclo (3.1.1) heptanes,6,6-dimethyl-2methylene-(1S)	0.09
5	46.128	Bicyclo (2.2.1) heptan-2-ol,1,7,7-trimethyl-acetate	0.18
6	45.827	Cyclohexane carboxylic acid, dimethyl phenyl ester	0.60
7	46.548	2,6,6-trimethylbicyclo (3.1.1) hepatane	0.57
8	46.606	2-Hexadecane,3,7,11,15-tetramethyl-(R-R^*∗*^, R^*∗*^-(E))	0.21
9	46.775	3-Tetradecyne	0.19
10	47.311	Hexadexanoic acid methyl ester	0.16
11	47.502	Cyclotetradecane	0.57
12	47.790	n-Hexadecanoic acid	17.17
13	48.411	Phytol	1.61
14	48.674	9,12-Octadecadienoic acid (Z, Z)	24.86
15	48.847	*cis*-13-Octadecadienoic acid	15.19
16	49.484	*cis*-13,16-Docasadienoic acid	5.29
17	50.042	1H- Indole	1.19
18	50.226	Imidazole,1-(9-borabicyclo (3.1.1) non-9-yl)	1.08
19	50.306	Hexadecanoic acid,2-hydroxy-1-(Hydroxymethyl) ethyl ester	1.51
20	52.295	Squalene	1.84
21	53.065	Tetracosane	0.57

**Table 6:** Biologically active chemical compounds of *JT*_AFI_- Hex extract.

S. No.	Retention time, min	Active components	Peak area %
1	42.509	Ar-Turmerone	0.85
2	42.748	Turmerone	0.35
3	44.348	Curlone (beta-turmerone)	0.52
4	46.555	Bicyclo (3.1.1) heptanes,6,6-dimethyl-,(1.alpha.,2.beta.,5.alpha)	0.32
5	47.686	n-Hexadecanoic acid	10.17
6	47.985	Cyclopentene,1-pentyl	0.89
7	48.060	1,19-Eicosadiene	1.09
8	48.412	Phytol	2.23
9	48.669	6-Octadecanoic acid	27.36
10	49.086	3-Dibenzofuranamine	13.24
11	49.435	Fumaric acid, 2, 4-dimethyl pent-3-yl isohexyl ester	5.36
12	49.503	Fumaric,3,5-dichlorophenyl isohexyl ester	4.99
13	49.666	Hentriacontane	5.67
14	50.124	Cis-13 = octadecenoic acid, methyl ester	1.16
15	50.258	Bezzoic anhyide, 4,4′,6,6′- Tetramethoxy -2,2′-dimethyl	2.68
16	50.428	Benzaldehyde, 6-Hydroxy-4-methoxy-2,3-dimethyl	1.65
17	50.573	4H-Pyran-4-one-2,6-diethyl-3,5-diemthyl	1.46
18	50.877	1-H-Imidazole-5-carboxamde,4-bebzoyl-N-(2-Pyridyl)	1.08
19	51.032	Lycopodan-5-1,12 hydroxy-15-methyl,(15 R)-	2.64
20	51.177	2,6-Dihydroxynaphthalene	1.97
21	51.335	Cylcopropaneoctanal,2-octyl-	1.00
22	51.438	9,12-Octadecadienoic acid (Z, Z)	3.12
23	52.292	2,6,10,14,18,22-Tetracosahexane	2.34
24	52.778	Stigmasterol	1.21
25	53.072	Tetracosane	0.82

**Table 7:** MIC values of *JT* extracts against reference bacterial strains.

MIC, mg/mL^*a*^							
Reference bacterial strains	Gram (±)	*JT*_YG_-EE	*JT*_YG_-Hex	*JT*_AFI_-EE	*JT*_AFI_-Hex	Gentamicin control	Streptomycin control
*S. aureus ATCC* 25923	+	7.81	7.81	15.60	31.25	≤0.0156	0.0019
*MRSA ATCC 33592* ^b^	+	15.60	31.25	15.60	31.25	ND^c^	ND
*S. epidermidis* RP62 A	+	1.95	1.95	15.60	31.25	0.012	≤0.0780
*E. faecalis ATCC 29212*	+	31.25	62.50	62.50	NI^d^	0.0039	0.0048
*E. coli ATCC 25 922*	−	62.50	NI	31.25	NI	0.0015	0.0039
*P. aeruginosa ATCC 27853*	−	15.60	NI	62.50	NI	0.2500	0.0078
*P. aeruginosa MDR ATCC BAA-2108* ^e^	−	NI	62.5	62.50	NI	ND	ND
*P. mirabilis ATCC 29245*	−	NI	NI	NI	NI	≤0.00003	0.0780
*K. pneumoniae ATCC 33495*	−	62.50	NI	62.50	NI	≤0.0007	0.0004

^*a*^MICs were determined by broth microdilution method and all experiments were done in triplicate; ^b^MICs were determined as 0.125 *µ*g/mL to ciprofloxacin and 1.5 *µ*g/mL to vancomycin; ^c^ND: not determined; ^d^NI: no inhibition; ^e^MICs were determined as > 32 *µ*g/mL to imipenem and ciprofloxacin.

**Table 8:** MIC values of *JT* extracts against clinical isolates of *S. aureus*.

MIC, mg/mL^*a*^							
Clinical isolates, *S. aureus*	*JT*_YG_- EE	*JT*_YG_- Hex	*JT*_AFI_- EE	*JT*_AFI_- Hex	Gentamicin control	Streptomycin control	Copper sulfate
704	1.95	7.81	7.81	62.50	≤0.0015	≤0.0004	0.00088
526	7.81	7.81	15.60	31.25	≤0.0015	≤0.0004	0.00088
250	15.60	7.81	15.60	62.50	≤0.0015	0.0078	0.00088
588	31.25	7.81	15.60	31.25	≤0.0015	0.0039	0.00088

^*a*^- MICs were determined by broth microdilution method and all experiments were done in triplicate.

## Data Availability

All data used during this study are available from the corresponding author.
